# Detection of aerobe–anaerobe mixed infection by metagenomic next-generation sequencing in an adult suffering from descending necrotizing mediastinitis

**DOI:** 10.1186/s12879-021-06624-4

**Published:** 2021-09-03

**Authors:** Jing Duan, Chuncheng Zhang, Xiaoshuang Che, Juanjuan Fu, Feng Pang, Qigang Zhao, Zhiqing You

**Affiliations:** 1 Department of Clinical Laboratory, Liaocheng People’s Hospital, No. 67, Dongchangxi Road, Dongchangfu District, Liaocheng, 252000 Shandong People’s Republic of China; 2 Department Hepatobiliary Surgery, Liaocheng People’s Hospital, No. 67, Dongchangxi Road, Dongchangfu District, Liaocheng, 252000 Shandong People’s Republic of China; 3 Department Computed Tomography, Liaocheng People’s Hospital, No. 67, Dongchangxi Road, Dongchangfu District, Liaocheng, 252000 Shandong People’s Republic of China

**Keywords:** Descending necrotizing mediastinitis, mNGS, Aerobe-anaerobe mixed infection

## Abstract

**Background:**

Descending necrotizing mediastinitis (DNM) is one of the most virulent forms of mediastinitis. The main causes of high mortality in DNM are believed to stem from difficulty and delay in the diagnosis. Fast and accurate identification of pathogens is important for the treatment of these patients. Metagenomics next-generation sequencing (mNGS) is a powerful tool to identify all kinds of pathogens, especially for rare and complex infections.

**Case presentation:**

A 64-year-old male patient was admitted to the intensive care unit (ICU) with unconsciousness, dyspnea, and swelling in the mandible and neck. Computed tomography (CT) scan results combined with clinical laboratory examination indicated DNM. Vancomycin and imipenem were used, and vacuum sealing drainage was applied for debridement and drainage of the infected area. The positive mNGS results of drainage fluid confirmed the presence of mixed infection caused by *Streptococcus anginosus, Prevotella oris*, and several other anaerobes. The antibiotics were adjusted to piperacillin/tazobactam and tinidazole according to the mNGS results and antimicrobial susceptibility testing of cultured pathogens. After 11 days of antibiotic therapy, the infection symptoms of the neck and mediastinum improved, and the patient was transferred out of the ICU on the 26^th^ day after negative result of drainage fluid culture.

**Conclusion:**

This case suggested that mNGS is a promising technology for precise and fast pathogens identification with high sensitivity, which may guide the diagnosis of infectious diseases in the future trend.

**Supplementary Information:**

The online version contains supplementary material available at 10.1186/s12879-021-06624-4.

## Background

Descending necrotizing mediastinitis (DNM) is a type of pyogenic mediastinitis, the infections usually have a fulminant course leading to sepsis and even death. Dental infection is a common cause of DNM, followed by retropharyngeal abscesses, peritonsillar abscesses, traumatic endotracheal intubation, trauma, cervical lymphadenitis, and osteomyelitis. Criteria for diagnosis of DNM are: (1) clinical manifestations of oropharyngeal infection; (2) characteristic roentgenographic features of mediastinitis; (3) documentation of mediastinitis during surgery or postmortem examination; (4) establishment of a relationship between oropharyngeal infection and subsequent necrotizing mediastinitis [1].The mortality of DNM has been reported to be 40%, which is approximately treble the risk of septic shock [2, 3]. However, prompt diagnosis, early aggressive incision, sufficient drainage, targeted antibiotic therapy combined with intensive care unit (ICU) management could significantly reduce the mortality to less than 20% [4]. Herein, we report a case in which mNGS was applied to identify the pathogens of DNM caused by odontogenic infection.

## Case presentation

**Fig. 1:**
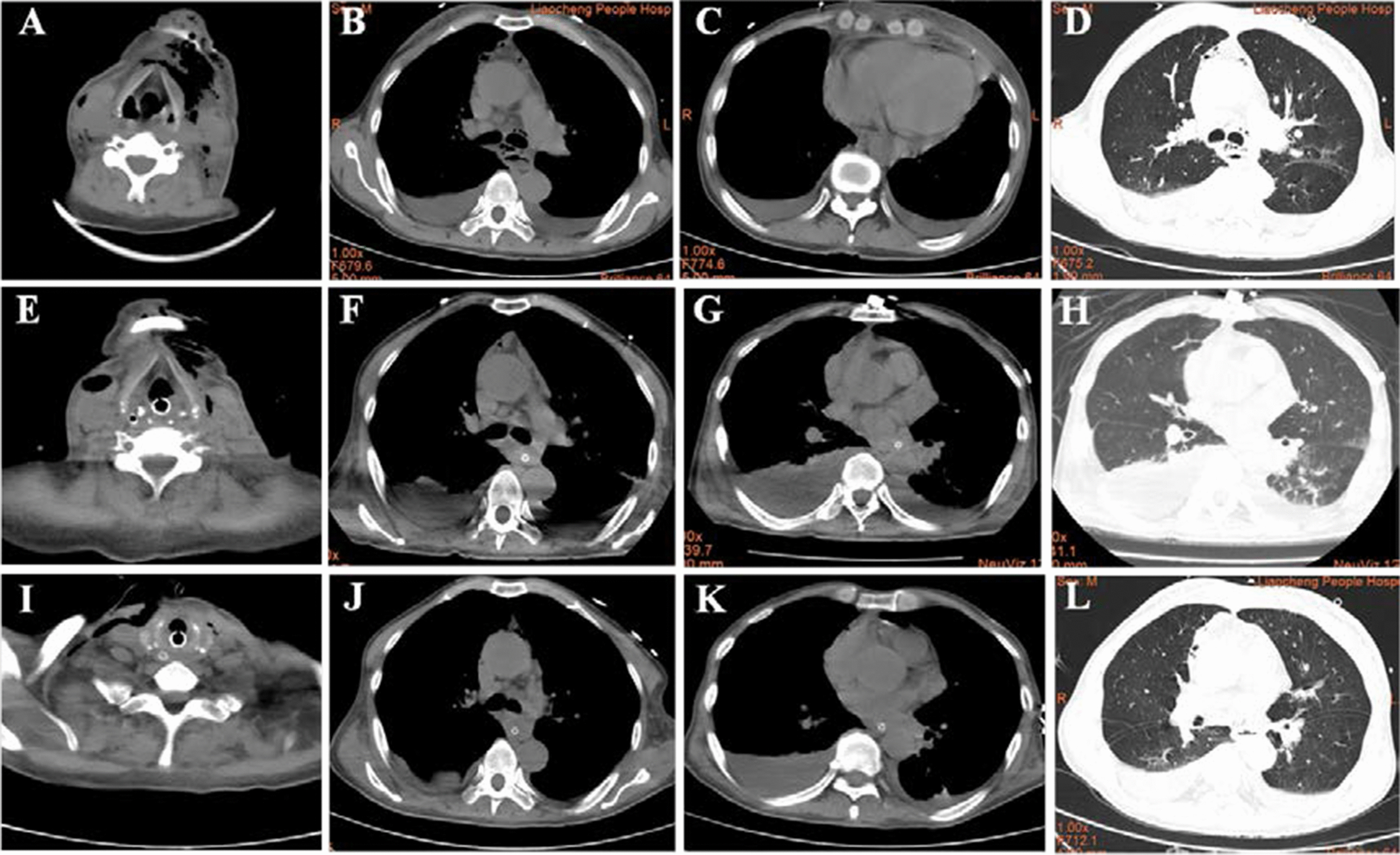
CT images of the patient at day 0, day 5 and day 11 of hospitalization.**A**–**D** The CT images of the patient at the time of admission, the mandibular space and mediastinum of the patient has obvious gas shadow, there is pleural effusion, and the lung image is normal; **E**–**H** The CT images of the patient 5 days after admission and treatment, the mandibular space and mediastinal gas after drainage treatment reduced, but the infection of the neck and mediastinum did not change significantly, the amount of pleural effusion increased compared with previous, and the lungs appeared as strips of increased density which indicating lung infection; **I**–**L** The CT images of the patient 11 days after admission, the submandibular space and mediastinal gas decreased, neck and mediastinal infections improved, lung infections improved, and pleural effusion decreased

A 61-year-old male patient with a history of hypertension was admitted to the ICU, with loss of consciousness and dyspnea. He had a toothache 15 days ago, and got some non-steroidal anti-inflammatory drugs in local pharmacy. However, the condition was not improved and mandible and neck begun to swell 10 days ago, and the situation deteriorated in the following days until he was admitted. The patient’s vitals were as follows: radial artery blood pressure was 112/77 mmHg, axillary temperature was 36.8℃, heart rate was 90 bpm, oxygen concentration was 45% with high flow oxygen inhalation, and blood oxygen saturation was 99%. Lung breath was clear, with no audible dry or wet rales. Blood test results: the leukocyte count was 4.05 × 10^9^ /L with high percentage of neutrophils and low percentage of lymphocyte (90.4% and 4.3%, respectively), C-reactive protein (CRP) was > 200 mg/L, and procalcitonin (PCT) was 9.32 ng/ml (Table 1). CT scan showed gas-like images in the left submandibular tissues and neck, cervical lymph node enlargement, obvious pleural effusion, and normal lungs (Fig. 1A–D). Clinical examinations and CT results indicated the presence of infection with aerogenic bacteria.

**Table 1:** Physical examination and laboratory test during the period of hospitalization

	Indexes	Day 0	Day 5	Day 11	Day 20
Physical examination	Axillary temperature: oxygen saturation: blood pressure: Status of affected area	36.8 ℃ 99% 112/77 mmHg red and swollen	37.3 ℃ 100% 135/72 mmHg red and swollen	36.5 ℃ 100% 128/65 mmHg red and swollen	36.7 ℃ 98% 114/68 mmHg swollen Improved
Laboratory test	WBC: (3.5–9.5) × 10^9^ /L Neutrophil%: (40–75) % CRP: (0–10) mg/L PCT: (0–0.5) ng/L IL-6: ≤ 5.4 pg/ml IL-10: ≤ 12.9 pg/ml	4.05 × 10^9^/L 90.4% > 200 mg/L 9.32 ng/ml 387.06 pg/ml 69.19 pg/m	5.77 × 10^9^ /L 87% > 200 mg/L 9.77 ng/ml 330.04 pg/ml 50.82 pg/m	4.61 × 10^9^ /L 69.4 19.79 mg/L 1.06 ng/ml 153.56 pg/ml 34.42 pg/m	7.53 × 10^9^ /L 70.5 NA NA 60.22 pg/ml 26.72 pg/ml

On the first day of hospitalization, the patient's blood oxygen saturation dropped to 80%, he was treated with tracheal intubation and mechanical ventilation, vacuum sealing drainage (VSD) was also applied for the debridement and drainage of the infected area. Symptomatic treatment was also conducted, such as nutritional support and maintenance of water-electrolyte balance. Vancomycin (VAN) was treated for suspected gram-positive cocci infection. On the second day of hospitalization, the inflammatory indexes were still high with wet rales in both lungs. Therefore, imipenem (IPM) was administered to treat gram-negative bacteria infection. However, on the third hospital day the temperature rose to 38.3℃, indomethacin suppository was administered rectally to bring down a fever. Drainage fluid from the lesions and blood were drawn to culture for microbes, an aliquot of the drainage fluid was also sent to the clinical laboratory for mNGS. On the 5^th^ day of hospitalization, the patient’s temperature returned to normal, but the percentage of neutrophils, CRP, and PCT levels were still high (87%, > 200 mg/L, 9.77 ng/mL, respectively). A second CT scan showed that the gas content in the tissues and extent of neck inflammation were reduced, but inflammatory lesions had appeared in the lungs, with increased bilateral pleural effusion volume (Fig. 1E–H). The result of mNGS revealed mixed infection by *Streptococcus anginous*, *Prevotella oris*, *Prevotella denticola*, *Peptostreptococcus stomatis*, *Fusobacterium nucleatum*, and *Alloprevotella tannerae* (Table 2). Thus, the antibiotic therapy was adjusted to piperacillin/tazobactam (TZP) and tinidazole (TNZ) based on positive results of mNGS.

**Table 2:** Pathogens recovered by mNGS in drainage fluid

Genus	Reads no.	Genus relative abundance	Species	Reads no.	Species relative abundance (%)
*Prevotella*	202,442	48.59%	*Prevotella oris*	78,656	18.32
			*Prevotella denticola*	27,149	8.53
*Streptococcus*	53,838	20.19%	*Streptococcus anginosus*	28,322	18.29
*Peptostreptococcus*	22,052	8.22%	*Peptostreptococcus stomatis*	21,470	8.09
*Fusobacterium*	11,837	4.53%	*Fusobacterium nucleatum*	6789	3.67
*Alloprevotella*	11,472	3.63%	*Alloprevotella tannerae*	11,347	3.58

**Fig. 2:**
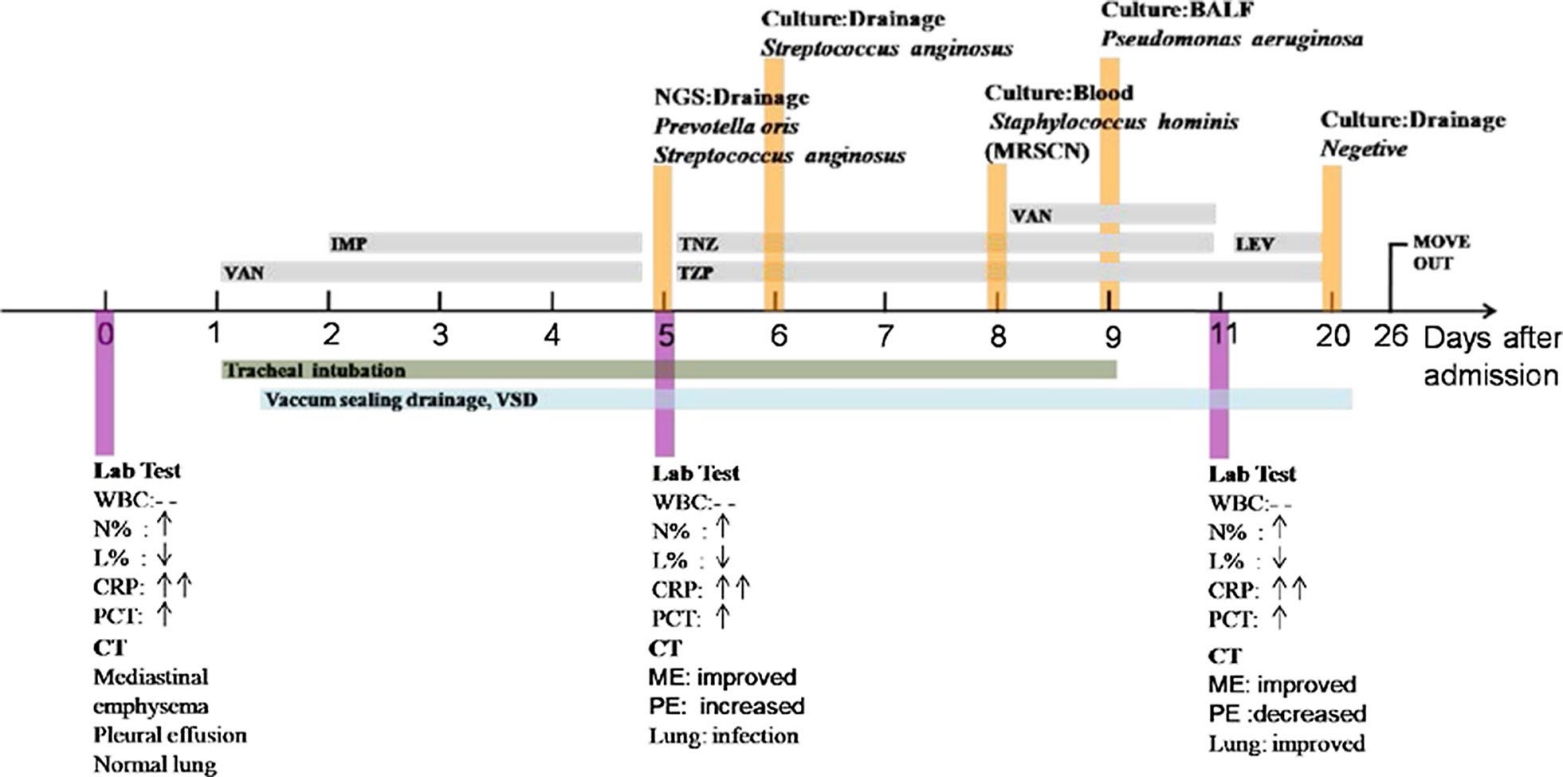
Schedule of lab test, diagnosis and therapy. --:normal, ↑ : above normal; ↑↑:beyond up limit; ↓ below normal

On the 6^th^ day of hospital stay, *Streptococcus anginous* was retrieved by culture of the drainage fluid, without any anaerobic bacteria detected. The isolated strain was sensitive to TZP (Table 3) according to antimicrobial susceptibility testing (AST) results. Hence, TZP and TNZ were continued for the treatment. The interpretive criterion of AST was established according to the latest edition of performance standards for antimicrobial susceptibility testing updated by the Clinical and Laboratory Standards Institute. Bronchoalveolar lavage was performed through fiberoptic bronchoscopy to remove secretions of respiratory, and bronchoalveolar lavage fluid (BALF) was sent to the microbiology laboratory for culture. On the 7th day of hospital stay, ultrasound-guided thoracic puncture was performed to drain pleural effusion fluids. On the 8th day of hospital stay, A methicillin-resistant coagulase-negative (MRSCN) *Staphylococcus hominis* strain was identified by blood cultures, and VAN was added to the antibiotic regiments according to the AST result (Table 3). On the 9th day a strain of *Pseudomonas aeruginosa* which sensitive to TZP was recovered from BALF by culture, thus change in antibiotics was not necessary (Fig. 2, Table 3). The patient’s general condition was improved and the mechanical ventilation was switched to high-flow oxygen inhalation. On the 11th day of hospital stay, the percentage of neutrophils returned to normal, CRP and PCT levels were significantly lower than before, indicating reduction of the infection. A third CT scan showed that the gas content in the inferior space and mediastinum cavity decreased, the infection symptoms of the neck and mediastinum relieved, the lung infection decreased, and the amount of pleural effusion was less extensively than before (Fig. 1I–L). Based on the patient’s improved clinical conditions and previous AST results, the antibiotic treatment was changed to Levaquin (LEV) and TZP. The patient was discharged out of ICU after obtaining a negative result of the drainage fluid bacterial culture (Fig. 2).

**Table 3:** Antimicrobial susceptibility testing results of cultured pathogens

Pathogen	Antimicrobial agent	Method (unit)	Interpretive standard					
			S	I	R	Result		Sensitivity
*Streptococcus anginosus*	Penicillin	MIC (ug/ml)	≤ 0.12	0.25–2	≥ 4	0.064		S
	Linezolid	KB (mm)	≥ 21	NA	NA	28		S
	Levofloxacin	KB (mm)	≥ 17	14–16	≤ 13	28		S
	Ceftriaxone	KB (mm)	≥ 27	25–26	≤ 24	30		S
	Vancomycin	KB (mm)	≥ 17	NA	NA	22		S
	Erythromycin	KB (mm)	≥ 21	16–20	≤ 15	6		R
	Azithromycin	KB (mm)	≥ 18	14–17	≤ 13	6		R
	Clindamycin	KB (mm)	≥ 19	16–18	≤ 15	6		R
	Clarithromycin	KB (mm)	≥ 21	17–20	≤ 16	6		R
	Tetracycline	KB (mm)	≥ 23	19–22	≤ 18	30		S
*Staphylococcus hominis*	Penicillin	MIC (ug/ml)	≤ 0.12	NA	≥ 0.25	≥ 0.50		R
	Vancomycin	MIC (ug/ml)	≤ 4	8–16	≥ 32	1.0		S
	Gentamicin	MIC (ug/ml)	≤ 4	8	≥ 16	1.0		S
	Clindamycin	MIC (ug/ml)	≤ 0.5	1–2	≥ 4	≥ 8.0		R
	Tetracycline	MIC (ug/ml)	≤ 4	8	≥ 16	≥ 16.0		R
	Ciprofloxacin	MIC (ug/ml)	≤ 1	2	≥ 4	1.0		S
	Levofloxacin	MIC (ug/ml)	≤ 1	2	≥ 4	0.5		S
	Moxifloxacin	MIC (ug/ml)	≤ 0.5	1	≥ 2	≤ 0.25		S
	Smz-tmp	MIC (ug/ml)	≤ 2/38	NA	≥ 4/76	80.0		R
	Rifampicin	MIC (ug/ml)	≤ 1	2	≥ 4	≤ 0.5		S
	Quinupristin/dalfopristin	MIC (ug/ml)	≤ 1	2	≥ 4	0.5		S
	Linezolid	MIC (ug/ml)	≤ 4	NA	≥ 8	2.0		S
	Erythromycin	MIC (ug/ml)	≤ 0.5	1–4	≥ 8	≥ 8.0		R
	Oxacillin	MIC (ug/ml)	≤ 0.25	NA	≥ 0.5	≥ 4.0		R
	Methicillin resistance							Positive
*Pseudomonas aeruginosa*	Piperacillin/tazobactam	MIC (ug/ml)	≤ 16/4	32/4–64/4	≥ 128/4	16.0		S
	Ceftazidime	MIC (ug/ml)	≤ 8	16	≥ 32	32.0		R
	Imipenenm	MIC (ug/ml)	≤ 2	4	≥ 8	≥ 16.0		R
	Meropenem	MIC (ug/ml)	≤ 2	4	≥ 8	≤ 0.25		S
	Amikacin	MIC (ug/ml)	≤ 16	32	≥ 64	≥ 64.0		R
	Ciprofloxacin	MIC (ug/ml)	≤ 0.5	1	≥ 2	0.5		S
	Levofloxacin	MIC (ug/ml)	≤ 1	2	≥ 4	1.0		S
	Cefoperazone/sulbactam	MIC (ug/ml)	≤ 16	32	≥ 64	32.0		I
	Cefepime	MIC (ug/ml)	≤ 8	16	≥ 32	16.0		I
	Tobramycin	MIC (ug/ml)	≤ 4	8	≥ 16	2.0		S
	Polymyxin	MIC (ug/ml)	≤ 2	NA	≥ 4	2.0		S
	Gentamicin	KB (mm)	≥ 15	13–14	≤ 12	17		S

## Discussion and conclusion

Acute purulent mediastinitis is a fatal condition which occurs after an esophageal perforation or that develops as a complication of odontogenic infection[5]. Infections in the posterior lower teeth are more likely to progress to DNM due to their drainage into the submandibular space, located near the retropharyngeal and lateral pharyngeal spaces[6]. Infection of the oropharyngeal cavity can rapidly spread along the fascial planes into the mediastinum space, where DNM develops. Because of the high lethality and rapid progression of DNM, prompt diagnosis, appropriate and adequate surgical drainage, in addition to supportive antibiotic therapy, combined with intensive medical care, are determinants for the success of the treatment and recovery [4]. CT reportedly contributes to early diagnosis of mediastinitis, owing to the capability of identifying the presence and extension of the disease, providing accurate information about the location and extent of the infection, guiding the management approach, and monitoring the drainage process [7, 8]. CT plays an important role in the diagnosis and assessment of therapeutic effectiveness. Another important factor that affects the clinical outcome of the patient is the type of microorganism that leading to the infection. As previously reported, DNM often comes from polymicrobial infection, and the most frequent microorganisms isolated are of the genus *Streptococcus* as well as anaerobic microbial bacteria of the genera *Prevotella*, *Peptostreptococcus*, *Bacteroides*, and *Fusobacterium*. Other pathogens isolated from DNM patients including bacteria belonging to the genera *Staphylococcus*, *Pesudomonas*, *Escherichia*, *Enterobacter*, *Acinetobacter*, *Enterococcus,* etc. [9, 10]. As reported in a literature review, a total of 156 microorganisms were isolated from 55 patients (61.8%), no pathogens can be isolated in approximately 40% of the cases of DNM [11]. The reason for this may be the complexity of the microorganisms and limitations of traditional microbial detection methods, such as morphological detection, culture, biochemical detection, and serotyping. These methods can only identify one or several specific pathogens, but cannot identify unknown or rare pathogenic microorganisms.

mNGS is a versatile technology which can identify pathogens more rapidly and precisely than traditional methods, and can even provide new insights into disease transmission, virulence, and antimicrobial resistance. Compared to traditional detection methods which can only detect certain targeted pathogens, mNGS is a shotgun sequencing method of RNA and DNA from clinical samples, where all DNA or RNA of the sample to be tested are mixed and sequenced, and the data is then compared with the pathogen database to obtain classification information of the pathogens. This method can detect tens of thousands of pathogens in a run within 48 h. The pathogen profiles include almost all viruses, bacteria, fungi, and parasites that can infect patients [12, 13].The details of materials and methods for mNGS was provided as supplement material (Additional file 1). Since 2013, professor Charles Chiu first applied mNGS to diagnose the encephalopathy caused by *Leptospira* infection in a 14-year-old boy [14], this method plays an increasingly important role in pathogen identification. As reported in previous studies, mNGS is more sensitive than culture, and is especially useful in the diagnosis of tuberculosis, fungi, viral and anaerobic bacteria infections [15]. This method is particularly important for the diagnosis of serious clinical infections, and intractable cases caused by complex pathogens. In this case, *Streptococcus anginosus* was cultured from drainage fluid, while other anaerobic pathogens were sterile by culture. However, mNGS retrieved all the pathogens presented in the infected tissues in a short time. The number of total reads obtained from the patient’s drainage fluid was 58,184,734, of which 407,293 reads were of microbial origin. *Prevotella oris* and *Streptococcus anginosus* were identified as the top two predominant pathogens, taking up 0.13% (78,656 reads) and 0.05% (28,322 reads) of the total sequence reads, and 19.31% and 6.95% of the total bacterial reads, respectively.

DNM is a serious infectious condition with aggressive and proliferative behavior which could be life-threatening. Prompt diagnosis, early surgical drainage, proper antibiotic therapy, and multidisciplinary collaborations are beneficial. Application of mNGS for pathogen detection makes it possible to detect all the microorganisms quickly, which is important for the adjustment of antibiotics. The wide application of mNGS would contribute to the characterization of pathogen profiles in DNM, which is still poorly characterized, since in almost 40% of the cases no pathogens can be detected by traditional methods. We believe that, with the progress of technological innovation and the decline of sequencing costs, mNGS will become a routine detection method for pathogenic microorganisms, and a growing number of patients will benefit from it.

## Supplementary Information

**Additional file 1.** Materials and methods for mNGS.

## Data Availability

The datasets analyzed during the current study are not publicly available due to patient privacy concerns but are available from the corresponding author on reasonable request.

## Abbreviations

DNM: Descending necrotizing mediastinitis
mNGS: Metagenomics next generation sequencing
CT: Computed tomographic
AST: Antimicrobial susceptibility testing
VSD: Vacuum Sealing Drainage,
CRP: C reaction protein
PCT: Procalcitonin
BALF: Bronchoalveolar lavage fluid
ME: Mediastinal emphysema
PE: Pleural effusion
IMP: Imipenem, VAN: vancomycin
TNZ: Tinidazole
TZP: Piperacillin/tazobactam
LEV: Levofloxacin
N%: Percentage of neutrophils
L%: Percentage of lymphocyte
