# Supplementary material for: Detection of aerobe–anaerobe mixed infection by metagenomic next-generation sequencing in an adult suffering from descending necrotizing mediastinitis
Source: BMC Infect Dis. 2021 Sep 3;21:905. doi: 10.1186/s12879-021-06624-4 (PMC8417974; doi:10.1186/s12879-021-06624-4)
Supplement: Supplementary file 1 — Additional file 1. Materials and methods for mNGS. [file 12879_2021_6624_MOESM1_ESM.docx]

**Additional materials S1: Materials and methods for mNGS**

**Materials and Methods**

Sample Processing and DNA Extraction

1.5-3mL sample from patient was collected according to standard procedures. 1.5mL microcentrifuge tube with 0.6mL sample and 250μL 0.5mm glass bead were attached to a horizontal platform on a vortex mixer and agitated vigorously at 2800-3200 rpm for 30 min. Then 7.2μL lysozyme was added for wall-breaking reaction. 0.3mL sample was separated into a new 1.5mL microcentrifuge tube, and DNA was extracted using the TIANamp Micro DNA Kit (DP316, TIANGEN BIOTECH) according to the manufacturer’s recommendation[1].

Construction of DNA libraries and Sequencing

Then, DNA libraries were constructed through DNA-fragmentation,end-repair, adapter-ligation and PCR amplification. Agilent 2100 was used for quality control of the DNA libraries. Qualified libraries were pooled,DNA Nanoball (DNB) was made and sequenced by BGISEQ-50 platform(**Table 4**)[2].

Bioinformatic analysis

High-quality sequencing data were generated by removing low-quality reads, followed by computational substraction of human host sequences mapped to the human reference genome (hg19) using Burrows-Wheeler Alignment[3]. The remaining data by removal of low-complexity reads were classified by simultaneously aligning to Pathogens metagenomics Database (PMDB), consisting of bacteria, fungi, viruses and parasites. The classification reference databases were downloaded from NCBI (ftp://ftp.ncbi.nlm.nih.gov/genomes/). RefSeq contains 4,945 whole genome sequence of viral taxa, 6,350 bacterial genomes or scaffolds, 1,064 fungi related to human infection, and 234 parasites associated with human diseases(**Table 4**).

Table S1. The timeline for completing mNGS

| Steps | Single Step | Time (min/hour) | In Total (hour) |
| --- | --- | --- | --- |
| DNA extraction | Inactivation at 56℃ | 30 min | 2 |
|  | Cell membranes breakdown (lyticase + bead-beat) | 30 min |  |
|  | Lysis (lysis buffer + Protease K） | 10 min |  |
|  | Magnetic bead adsorption(bead +isopropanol) | 10 min |  |
|  | Wash 3 times | 10 min |  |
|  | DNA elution | 10 min |  |
|  | DNA concentration quantification | 15 min |  |
| Library construction | Digestion | 20 min | 3 |
|  | Magnetic beads purification | 20min |  |
|  | End joining repair | 25 min |  |
|  | DNA connection | 20 min |  |
|  | Magnetic beads-based purification | 20 min |  |
|  | PCR amplification | 40 min |  |
|  | Magnetic beads purification | 20 min |  |
| DNB preparation | Library quantification | 15 min | 1.5 |
|  | Pooling | 5 min |  |
|  | Single-stranded circles formation | 6 min |  |
|  | Circularization | 30 min |  |
|  | DNB1 | 5 min |  |
|  | DNB2 | 15 min |  |
|  | DNB concentration quantification | 15 min |  |
| Sequencing | Loaded and sequenced | 15 h | 15 |
| Bioinformatics analysis | Data transmission&Bioinformatics analysis | 2 h | 2.0 |
| Report interpretation | Report interpretation | 0.5 h | 0.5 |
| In Total |  |  | 24 |

**References**

1. Long Y, Zhang YX, Gong YP, Sun RX, Su LX, Lin X, et al. Diagnosis of Sepsis with Cell-free DNA by Next-Generation Sequencing Technology in ICU Patients. Archives of Medical Research. 2016; 47(5): 365-371. <https://doi.org/10.1016/j.arcmed.2016.08.004>.
2. Jeon, YJ, Zhou YL, Li YH, Guo QW, Chen JC, Quan SM, et al. The feasibility study of non-invasive fetal trisomy 18 and 21 detection with semiconductor sequencing platform. PLoS One, 2014. 9(10): p. e110240.https://doi.org/10.1371/journal.pone.0110240.
3. Li H, R Durbin, Fast and accurate short read alignment with Burrows-Wheeler transform. Bioinformatics, 2009. 25(14): 1754-1760.https://doi.org/10.1093/bioinformatics/btp324
